# Syncope as the Initial Presentation of Takayasu Arteritis in a 57‐Year‐Old Female: A Case Report and Literature Review

**DOI:** 10.1002/ccr3.72946

**Published:** 2026-06-15

**Authors:** Chao Liu, Jiale Gan, Lili Feng, Yang Yang, Lianwei Dong, Jiabao Liu, Dingguo Zhang

**Affiliations:** ^1^ Ningxia Hui Autonomous Region Hospital of Traditional Chinese Medicine Ningxia Hui Autonomous Region Academy of Traditional Chinese Medicine Yinchuan Ningxia Province China; ^2^ Department of Cardiovascular Medicine The First Affiliated Hospital of Nanjing Medical University Nanjing Jiangsu Province China

**Keywords:** chronic inflammatory disorder, immunosuppressive therapy, large vessel vasculitis, syncope, Takayasu arteritis

## Abstract

Takayasu arteritis (TA) is a rare chronic granulomatous large‐vessel vasculitis predominantly affecting the aorta and its major branches, leading to stenosis, occlusion, or aneurysm formation. It typically presents in young women, though atypical cases can occur in older adults. Diagnosis is often delayed owing to nonspecific symptoms and a lack of reliable biomarkers. We report a case of a 57‐year‐old woman who presented with recurrent syncope, dizziness, and chest tightness. Physical examination revealed a significant blood pressure discrepancy between arms and carotid bruits. Although erythrocyte sedimentation rate (ESR) was normal, other inflammatory markers were elevated. Angiography and computed tomography angiography demonstrated multi‐vessel involvement, including subtotal occlusion of the right subclavian artery, complete occlusion of the left subclavian artery, and stenosis of the right vertebral artery and abdominal aorta. The patient met the 2022 American College of Rheumatology classification criteria for TA. Treatment with glucocorticoids and cyclophosphamide resulted in symptomatic improvement, with no recurrence during a six‐month follow‐up. This case underscores that syncope can be a rare initial manifestation of TA, and normal ESR does not exclude active disease. Thorough clinical evaluation and vascular imaging are essential for timely diagnosis and management to prevent serious complications.

## Key Clinical Message

Takayasu arteritis should be considered in any patient with syncope and asymmetric pulses or inter‐arm blood‐pressure differences, even if the erythrocyte sedimentation rate is normal. Careful physical examination and vascular imaging are essential, and prompt glucocorticoid and immunosuppressive therapy can relieve symptoms and prevent progression.

## 1 Introduction

Takayasu arteritis (TA) is a chronic immune‐mediated granulomatous vasculitis of unknown etiology that primarily involves the aorta and its major branches, leading to wall thickening, fibrosis, stenosis, occlusion, thrombosis, and aneurysmal dilatation [1, 2, 3]. It has a worldwide distribution but predominates in Asia and chiefly affects women between 10 and 40 years of age, with reported female‐to‐male ratios of 1.2:1 to 29:1 [4, 5]. The pathogenesis is multifactorial, involving genetic, autoimmune, environmental, and hormonal influences, with mast cells and cytotoxic lymphocytes driving granulomatous mural inflammation [6, 7, 8, 9]. Because symptoms are nonspecific, serological biomarkers are unreliable, and clinical manifestations are heterogeneous, TA is frequently under‐recognized or diagnosed late, delaying treatment [10, 11].

Diagnosis, therefore, relies on careful clinical assessment, inflammatory markers (ESR and C‐reactive protein), and vascular imaging such as angiography, CTA, MRI, ultrasonography, or PET [12, 13]. Management is multidisciplinary and primarily comprises glucocorticoids and immunosuppressive agents, with endovascular or surgical intervention reserved for critical ischemia from irreversible stenosis [14, 15]. Although neurological involvement in TA is well recognized, syncope as the initial and isolated presenting symptom is exceedingly rare and has been documented mainly in younger patients with concurrent constitutional features [16]. We report a 57‐year‐old woman whose sole initial presentation was syncope, without prodromal or systemic inflammatory symptoms, and despite a normal ESR with active multi‐territory vascular inflammation—a combination, to our knowledge, rarely documented, illustrating the diagnostic challenges of such atypical presentations.

## 2 Case Presentation

### 2.1 Clinical History and Physical Examination

A 57‐year‐old woman presented for evaluation of two syncopal episodes during the past year, each characterized by sudden, transient loss of consciousness lasting approximately 10 s, with spontaneous recovery and without a prodrome of nausea, diaphoresis, or palpitations. Neither episode was associated with witnessed convulsions, tongue biting, or post‐ictal confusion. Both events occurred while the patient was in an upright position during routine daily activities. There was no preceding orthostatic change reported, and no triggers such as coughing or micturition were identified. The patient had no recall of the events themselves, though bystanders reported a brief collapse followed by rapid return to full consciousness. Experienced symptoms of chest tightness and dizziness in the past month.

The patient denied any history of fever, tussis, dyspnea, heart palpitations, eructation, acid reflux, tinnitus, nausea, emesis, abdominal discomfort, or motor abnormalities. The patient denied any history of diabetes, hypertension, coronary artery disease, asthma, thyroid disease, or pulmonary tuberculosis. The patient denied use of hormonal therapy, oral steroids, and traditional Chinese medicine. At admission, the patient was afebrile (36.5°C) with a heart rate of 72 bpm, respiratory rate of 18/min, and oxygen saturation of 95% on ambient air. A significant blood pressure discrepancy was noted between the right and left arms. Blood pressure measured in the right arm in the sitting position was 120/70 mmHg, but in the left arm in the sitting position, it was 100/50 mmHg. The pulse at the left radial artery was weak, while other peripheral pulses were palpable. Bilateral carotid bruits were audible. The patient was alert and lucid with fluent speech, pupils equal and round bilaterally, and reactive to light. Muscle strength and tone were normal in all four limbs. Deep tendon reflexes, including the biceps and triceps jerks in the upper limbs and the patellar and Achilles tendon reflexes in the lower limbs, were normoactive and symmetric. Both the plantar reflexes were flexor. Babinski's and Oppenheim's signs were absent. Meningeal signs were negative, with no nuchal rigidity, Kernig's sign, or Brudzinski's sign.

### 2.2 Laboratory Tests and Radiological Investigations

Her hematological, biochemical, serological, coagulation function, and autoimmune tests revealed normal findings. Her erythrocyte sedimentation rate (ESR) was 12 mm/h (reference range: 0–20 mm/h for females), which was within normal limits. High‐sensitivity C‐reactive protein (hs‐CRP) was elevated at 7.08 mg/L (reference range: < 3.0 mg/L). Interleukin‐6 (IL‐6) was also elevated at 12.28 pg/mL (reference range: < 7.0 pg/mL). The patient was admitted due to recurrent syncope, chest tightness, and dizziness. Coronary angiography is indicated to evaluate for possible coronary artery stenosis and guide further management.

**FIGURE 1:**
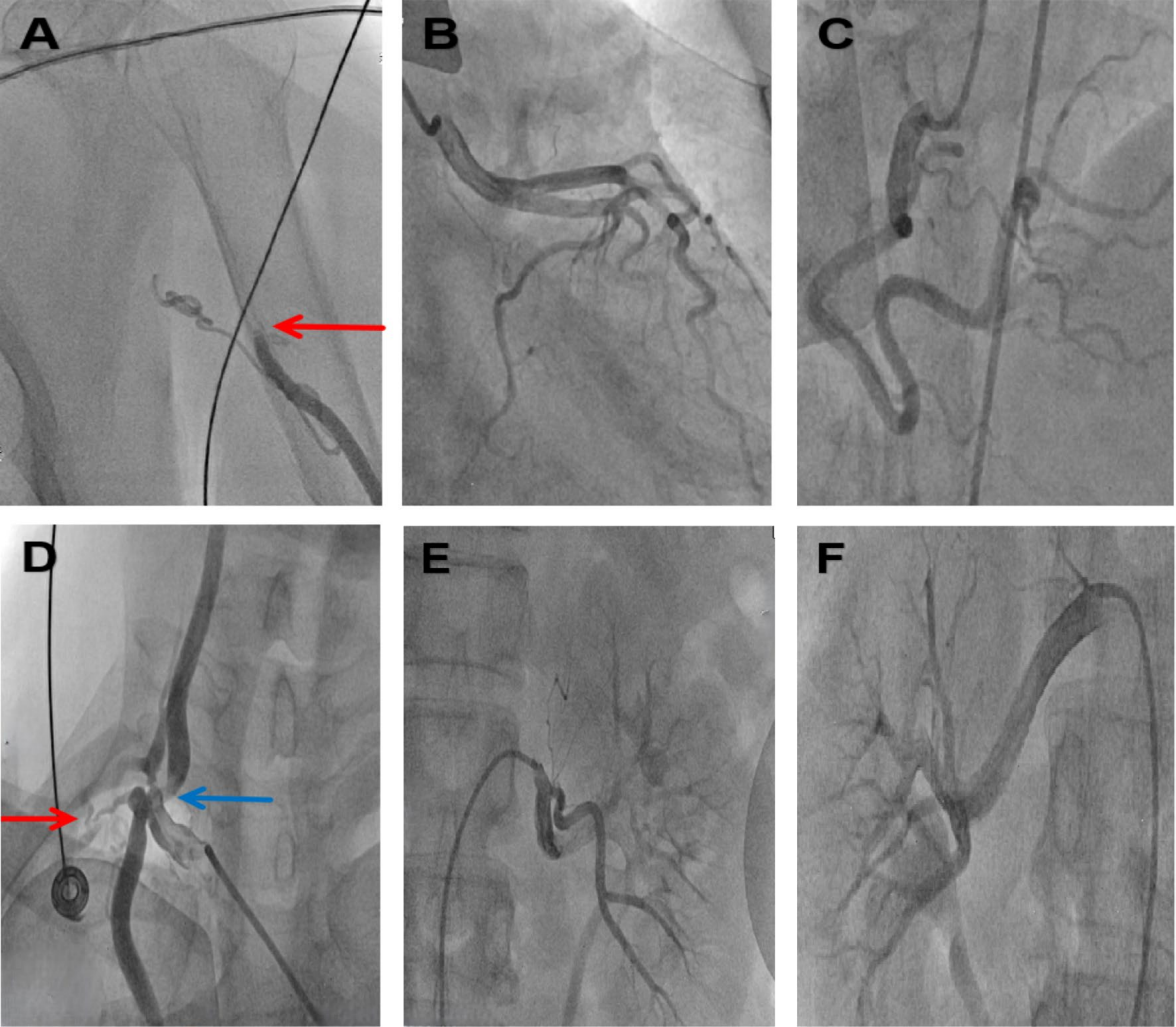
(A) Left brachial artery angiography showing occlusion of the proximal left brachial artery, indicated by a red arrow; (B) Coronary angiography demonstrating no obvious stenosis in the left coronary system; (C) Coronary angiography demonstrating no obvious stenosis in the right coronary artery; (D) Moderate‐to‐severe stenosis at the ostium of the right vertebral artery, indicated by a blue arrow, and subtotal occlusion of the right subclavian artery, indicated by a red arrow; (E) Left renal artery without stenosis; (F) Right renal artery without stenosis.

During the procedure, right radial artery access was primarily attempted. Following successful cannulation, advancement of the coronary angiography catheter to the right subclavian artery proved difficult. Angiography revealed right subclavian artery subtotal occlusion. Subsequently, left radial access was established without complication. However, the catheter encountered resistance at the level of the left brachial artery. Selective angiography confirmed occlusion of the proximal left brachial artery (Figure 1A). Ultimately, right femoral arterial access was utilized, enabling successful coronary angiography. Findings demonstrated: Left anterior descending artery and circumflex artery: No stenosis (Figure 1B). Right coronary artery: No stenosis (Figure 1C). During the procedure, we suspected the possibility of large‐vessel vasculitis. Later, the JR 3.5 angiographic catheter was inserted into the innominate artery, and subsequent selective angiography demonstrated: a moderate‐to‐severe stenotic lesion at the ostium of the right vertebral artery (Figure 1D), non‐visualization of the left common carotid and left subclavian arteries despite repeated contrast injections. Renal artery angiography showed the left renal artery without stenosis (Figure 1E) and the right renal artery without stenosis (Figure 1F).

On initial evaluation, the differential diagnosis for recurrent syncope in this patient included: (1) cardiac causes, specifically arrhythmia and coronary artery disease—given her age and the complaint of chest tightness—which prompted coronary angiography; (2) orthostatic hypotension; (3) vasovagal syncope; and (4) cerebrovascular disease, given the presence of bilateral carotid bruits and dizziness. Large‐vessel vasculitis was not the primary suspicion at admission; however, the incidental finding of subclavian artery occlusion during coronary angiography, combined with the physical examination findings (inter‐arm blood pressure discrepancy, diminished left radial pulse, bilateral carotid bruits), prompted urgent consideration and further evaluation for Takayasu arteritis. During this admission, further diagnostic studies were conducted to exclude other vasculitic processes, such as antinuclear antibody (ANA), ribonucleoprotein (RNP), anti‐CCP antibody, double‐stranded DNA, centromere antibody, histone, histidyl tRNA synthetase (JO‐1), topoisomerase 1 (SCL‐70), Sjögren's‐syndrome‐related antigen A (SSA), and Sjögren's‐syndrome‐related antigen B (SSB) autoantibodies, rheumatoid factor, C‐ANCA and P‐ANCA, anti‐DSDNA, and anti‐SS were negative. immunoglobulin levels (IgA, IgM, and IgG) and C3, C4 complement levels were within normal limits. Anti‐cardiolipin total antibody (IgA/G/M), antiphospholipid antibody (IgA, IgM, and IgG) levels remained normal. Her infectious disease screening was negative, encompassing tests for hepatitis B and C, human immunodeficiency virus, Epstein–Barr virus, syphilis, and respiratory viral pathogens.

**FIGURE 2:**
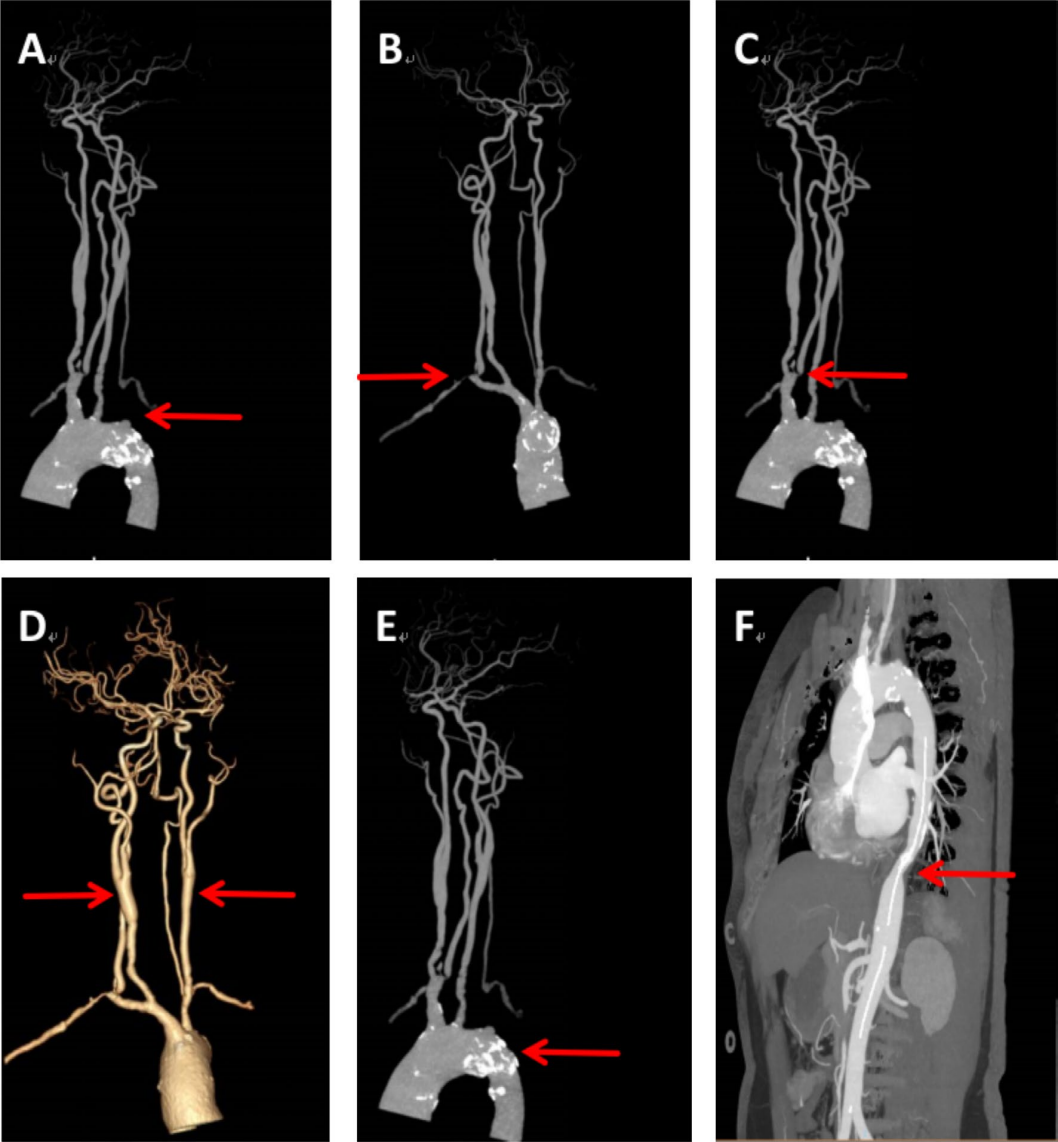
(A) Complete occlusion of the proximal left subclavian artery proximal to the origin of the left vertebral artery, with beaded appearance of the vessel wall indicated by a red arrow; (B) Subtotal occlusion of the proximal right subclavian artery lumen indicated by a red arrow; (C) Moderate‐to‐severe origin stenosis of the right vertebral artery indicated by a red arrow; (D) Bilaterally symmetric circumferential wall thickening with beaded appearance in the proximal common carotid arteries, associated with distal aneurysmal dilatation indicated by a red arrow; (E) Concentric wall thickening with calcification in the thoracic aorta indicated by a red arrow; (F) Wall thickening with luminal narrowing in the abdominal aorta indicated by a red arrow.

We also performed head–neck CTA examinations on the patients, and the examination results showed complete occlusion of the proximal left subclavian artery proximal to the origin of the left vertebral artery, with a beaded appearance of the vessel wall (Figure 2A). Subtotal occlusion of the proximal right subclavian artery lumen (Figure 2B). Moderate‐to‐severe origin stenosis of the right vertebral artery was observed (Figure 2C). Bilaterally symmetric circumferential wall thickening with a beaded appearance was located at the proximal common carotid arteries, associated with distal aneurysmal dilatation (Figure 2D). The thorax and abdomen CTA showed concentric wall thickening with calcification in the thoracic aorta (Figure 2E). Wall thickening with luminal narrowing was observed in the abdominal aorta (Figure 2F).

The imaging findings were pivotal in confirming the diagnosis of Takayasu arteritis. The key diagnostic features on CTA included: (1) complete occlusion of the proximal left subclavian artery with a characteristic beaded appearance of the vessel wall, indicating mural inflammation rather than atherosclerosis; (2) subtotal occlusion of the proximal right subclavian artery; (3) bilaterally symmetric circumferential wall thickening with beading in the proximal common carotid arteries, with associated distal aneurysmal dilatation—a pattern highly characteristic of TA; (4) moderate‐to‐severe stenosis of the right vertebral artery origin; and (5) concentric wall thickening with calcification in both the thoracic and abdominal aorta with luminal narrowing. The symmetric, circumferential, and multi‐territory pattern of mural thickening, involving both the aorta and its major branches, was most critical in differentiating inflammatory vasculitis from atherosclerotic disease. Among these, the bilaterally symmetric circumferential wall thickening with beaded appearance involving the proximal common carotid arteries, combined with multi‐territory involvement (subclavian, vertebral, thoracic, and abdominal aorta), was the most critical imaging feature distinguishing Takayasu arteritis from atherosclerotic disease.

According to the Numano classification system, our patient was classified as Type V, reflecting extensive involvement of the aortic arch and its branches (bilateral subclavian arteries, bilateral common carotid arteries, and right vertebral artery), the descending thoracic aorta, and the abdominal aorta.

### 2.3 Diagnostic Strategy

According to the 2022 revised American College of Rheumatology criteria for Takayasu arteritis. Absolute requirements for the diagnosis of TAK include an age of onset prior to the age of 60 years and evidence of vasculitis on imaging [17]. Other points‐based criteria include the following: female sex (+1), presence of ischemic chest pain (+2), intermittent claudication (+2), vascular bruit (+2), diminished upper extremity pulses (+2), diminished carotid pulse or tenderness to palpation (+2), an inter‐arm blood pressure difference of ≥ 20 mmHg (+1), the number of affected arterial beds (+1 to +3), bilateral involvement of the arteries (+1), and abdominal aortic involvement with extension to the renal or mesenteric arteries (+3). A diagnosis of TA was established in patients with a cumulative score of ≥ 5 points. Based on the physical examination and radiological findings, our patient fulfilled the new diagnostic criteria for TA with a total score of nine. Points were assigned as follows: female (+1), carotid bruit (+2), reduced radial pulse in the left upper limb (+2), systolic blood pressure difference of 20 mmHg between both arms (+1), and involvement of the aortic arch and abdominal aorta (+2), bilateral subclavian artery involvement (+1). The diagnostic cutoff is 5 points. Our case fulfilled the American College of Rheumatology criteria for TA [17].

The patient was a 57‐year‐old woman at the time of initial diagnosis. Her condition had an insidious onset with an absence of constitutional symptoms. Clinical detection was markedly delayed despite extensive vascular involvement affecting multiple major aortic branches and a highly atypical progression pattern. Rheumatology services were consulted. Given the active peri‐aortic inflammation, which conferred an elevated procedural risk, endovascular intervention was deemed temporarily contraindicated. She was treated in the line of TA with oral prednisolone 15 mg twice a day, aspirin 100 mg once daily, plus calcium carbonate tablets one tablet once daily orally. A follow‐up rheumatology visit was arranged 2 weeks after discharge to evaluate therapeutic efficacy and adjust management accordingly.

### 2.4 Treatment and Follow‐Up

Following the two‐week post‐discharge review, at which the patient remained symptomatic with persistent dizziness and chest tightness on prednisolone 30 mg/day, intravenous cyclophosphamide was initiated as a steroid‐sparing immunosuppressive agent. Cyclophosphamide was administered intravenously on a biweekly schedule with a tapering dose protocol: 0.6 g for the first two infusions, followed by 0.4 g for the next two, and then 0.2 g for the final two infusions—spanning a total of 3 months. Oral prednisolone was tapered concurrently. By the fourth month, cyclophosphamide was discontinued, and the patient was maintained on prednisolone 5 mg/day with aspirin 100 mg/day and calcium carbonate supplementation. At the six‐month follow‐up, the patient had experienced no further syncopal episodes. Dizziness and chest tightness had significantly improved. Inflammatory markers showed normalization of hs‐CRP. No new vascular events occurred. Repeat vascular imaging was planned at 12 months to assess for disease progression or new lesions. The patient tolerated the treatment well, without significant adverse effects.

## 3 Discussion

Takayasu arteritis is a granulomatous vasculitis of medium‐sized and large arteries characterized by transmural fibrous thickening, occlusion, and ischemia, in which early diagnosis is frequently delayed [18]. Although historically regarded as a disease of young East Asian women, cases have been reported across a wide age range [19]. The pathogenesis is multifactorial, involving genetic susceptibility, autoimmune mechanisms, and potential triggers such as *Mycobacterium tuberculosis* infection [20], with panarteritis progressing to stenosis, occlusion, and arterial dilation [21]. Diagnosis integrates clinical, imaging, and serological findings [22], and physical examination remains pivotal: asymmetric pulses, inter‐arm blood pressure discrepancy, and bruits over the great vessels are present in over 90% of cases—features of low sensitivity but high specificity that guide further investigation [13].

In our patient, giant cell arteritis, infectious arteritides (syphilis, tuberculosis), connective tissue disorders with large‐vessel involvement, and polyarteritis nodosa were excluded by laboratory testing; polyarteritis nodosa was additionally inconsistent with the multi‐territorial large‐vessel distribution, since it characteristically spares the aorta and major elastic arteries. Active vasculitis may persist despite normal CRP and ESR, and overreliance on ESR (sensitivity 72%, specificity 56%) may delay treatment; diagnostic delays of several years are common owing to nonspecific presentation, absent biomarkers, and limited clinician awareness [23]. Syncope as the initial manifestation of TA is exceptionally rare; Table 1 summarizes nine such cases, most in younger patients with elevated inflammatory markers and established disease, in whom syncope followed known carotid or vertebral involvement rather than preceding it. Although our patient's clinical course was indolent and ESR remained within the normal range, the elevation of hs‐CRP and IL‐6, together with circumferential mural thickening with a beaded appearance on CTA and peri‐aortic inflammatory changes, indicates that this case represented active TA with a falsely normal ESR, rather than the burned‐out, chronic fibrotic phase. In such cases, structural vascular damage contributes to symptoms through hemodynamic compromise, but subclinical inflammation captured by CRP and IL‐6, and not by ESR, continues to drive disease activity.

**TABLE 1:** Systematic comparison of published cases of Takayasu arteritis presenting with syncope.

Author (year)	Age/Sex	Syncope mechanism	ESR/CRP	Key vessels involved	Diagnostic route	Treatment
Khadka et al. (2022) [24]	26F	Bilateral subclavian occlusion, cerebral hypoperfusion	ESR 51 mm/h; CRP 28.1 mg/L (both elevated)	Bilateral subclavian, carotid, and brachiocephalic arteries	MRI angiography	Prednisolone, MTX, aspirin
Peera et al. (2011) [25]	48F	SSS, vertebrobasilar insufficiency	Not reported	Subclavian artery	Emergency Dept evaluation; DSA	Steroids
Torere et al. (2023) [26]	34F (Caucasian)	SSS via left subclavian occlusion, vertebrobasilar insufficiency	ESR 66 mm/h (elevated); CRP 1.5 mg/L	Left subclavian; aorta; left common carotid (> 90% stenosis)	ED presentation; CTA neck	Steroids Methotrexate
Menon & Himabindu (2010) [27]	19F (Asian)	Orthostatic hypoperfusion (convulsive syncope)	ESR 42 mm/h; CRP 26 mg/L (both elevated)	Bilateral carotid, left subclavian	Neurology referral; Doppler; DSA	Prednisolone 40 mg/day
Pallangyo et al. (2020) [28]	17 M (African)	Total occlusion of the left carotid/vertebral, cerebral ischemia (convulsive syncope)	ESR 58 mm/h; CRP 27.6 mg/L (both elevated)	Left CCA, ICA, ECA, vertebral artery (total occlusion)	Neurology referral; MRA brain	Dexamethasone MTX
[29]	28F (pregnant, 28 week)	Bilateral upper limb pulselessness, convulsive syncope (refractory to anticonvulsants)	ESR 40 mm/h; CRP elevated	Bilateral upper limb arteries; aorta branches	Obstetric emergency; angiography	Steroids antihypertensives
Ducas‐Mowchun et al. (2023) [30]	29F	Critical bilateral carotid/subclavian narrowing, posterior circulation hypoperfusion	ESR/CRP: normal	Left CCA, right subclavian; thoracic aorta	Internal medicine; CT angiography	Glucocorticoids; immunosuppressants
Choi et al. (2023) [31]	50F	Left subclavian total occlusion, SSS during arm elevation (provoked syncope)	ESR 43 mm/h (elevated); CRP 1.20 mg/L	Left subclavian (total occlusion); right CIA stenosis	CTA; endovascular PTA	PTA rheumatology medical Rx
Ahamed et al. (2025) [32]	77 M (elderly, learning disability)	Aortic arch branch vessel disease, global cerebral hypoperfusion	ESR 83 mm/h; CRP 136 mg/L (both elevated)	Aorta; arch vessels (circumferential thickening)	Inpatient workup; imaging	Corticosteroids

Abbreviations: CCA, common carotid artery; CIA, Common iliac artery; CRP, C‐reactive protein; CTA, computed tomography angiography; DSA, digital subtraction angiography; ECA, external carotid artery; ED, emergency department; ESR, erythrocyte sedimentation rate; F, female; ICA, internal carotid artery; M, male; MRA, magnetic resonance angiography; MRI, magnetic resonance imaging; MTX, methotrexate; PTA, percutaneous transluminal angioplasty; SSS, subclavian steal syndrome.

The patient fulfilled the 2022 ACR revised classification criteria for TA, supported by bilateral subclavian artery occlusions, diffuse arterial wall thickening, and multi‐territorial involvement of the carotid, vertebral, and descending aortic vessels; the absence of atherosclerotic risk factors, an elevated hsCRP, and exclusion of mimickers further confirmed inflammatory vasculitis. The most plausible mechanism of syncope in our patient is vertebrobasilar insufficiency arising from a dual posterior‐circulation insult: subclavian steal physiology on the left, where the proximal left subclavian artery was completely occluded proximal to the origin of the left vertebral artery, together with a moderate‐to‐severe ostial stenosis of the right vertebral artery. Concomitant bilateral common carotid wall thickening may have further reduced collateral capacity. The absence of orthostatic blood‐pressure change, cardiac arrhythmia, or coronary disease makes global hypoperfusion of cardiac or autonomic origin unlikely. Limited provocative imaging (e.g., arm‐exercise transcranial Doppler) before therapy is acknowledged as a limitation.

The 2018 EULAR recommendations advocate combined corticosteroid and immunosuppressive therapy for early remission induction, with prednisolone as the principal agent and adjunctive immunosuppressants (azathioprine, methotrexate, mycophenolate mofetil, cyclophosphamide, or leflunomide) or biologic agents (infliximab, tocilizumab, or etanercept) added to limit corticosteroid‐related complications and reduce relapse [33]. Because active peri‐aortic inflammation conferred elevated procedural risk, endovascular intervention was temporarily contraindicated; the patient was therefore started on oral prednisolone 15 mg twice daily, aspirin 100 mg daily, and calcium carbonate, with rheumatology review arranged at 2 weeks.

At review, persistent dizziness and chest tightness without recurrent syncope indicated a suboptimal response to high‐dose prednisolone monotherapy. Given the chronic, relapsing nature of TA and the need for a steroid‐sparing strategy, biweekly intravenous cyclophosphamide was added in accordance with the EULAR remission‐induction strategy [34]. Combined therapy thus enabled early intervention; comprehensive clinical evaluation supported by multimodal vascular imaging remains essential to mitigate disease progression and prevent the life‐threatening complications of Takayasu arteritis.

## 4 Conclusion

This case demonstrates that syncope can be a rare but clinically significant initial manifestation of Takayasu arteritis, even in older patients without constitutional symptoms. Critically, a normal ESR does not exclude active disease, as illustrated by our patient's elevated hs‐CRP and IL‐6 alongside circumferential mural thickening on CTA. The underlying mechanism in this case was vertebrobasilar insufficiency resulting from combined subclavian steal physiology and right vertebral artery stenosis. Meticulous physical examination, particularly the detection of inter‐arm blood pressure discrepancy, diminished peripheral pulses, and vascular bruits, together with multimodal vascular imaging, remain essential for timely diagnosis. Prompt initiation of glucocorticoid and immunosuppressive therapy can achieve symptomatic remission and prevent life‐threatening vascular complications.

## Author Contributions

**Chao Liu:** resources, validation, writing – original draft. **Jiale Gan:** writing – review and editing. **Lili Feng:** supervision. **Yang Yang:** resources. **Lianwei Dong:** validation. **Jiabao Liu:** supervision, validation. **Dingguo Zhang:** conceptualization, project administration, resources, supervision, writing – review and editing.

## Funding

The author(s) declare that financial support was received for the research, authorship, and/or publication of this article. This work was supported by the National Natural Science Foundation of China (81901416) and the Natural Science Foundation of Jiangsu Province (BK20191067).

## Ethics Statement

The study was conducted in accordance with the ethical principles of the Declaration of Helsinki.

## Consent

Informed written consent was obtained from the patient for publication of this report and any accompanying images.

## Conflicts of Interest

The authors declare no conflicts of interest.

## Data Availability

All raw data and code are available upon request.
